# Bridging the gap… From clinical research to clinical practice

**DOI:** 10.4103/0972-0707.43409

**Published:** 2008

**Authors:** Anil Kohli

**Affiliations:** President, Dental Council of India

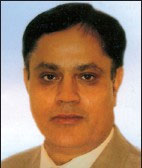



The purpose of clinical research is to enhance the transfer of science into clinical practice. The status of our specialty research will foretell the future effectiveness and scope of the services provided. Dental research, including how it is presented, communicated, and validated, is perhaps the single most important component that establishes the future success of the specialties of oral and dental sciences. All dentists have a vital stake in research, as the wellspring of their personal professional future. Through facilitating advancing the knowledge and technological base of clinical practice, research offers a dental surgeon the opportunity to transfer ideas into clinical research.

Hence, concerns over the vitality and sustainability of the research profession deserve detailed attention and understanding of every dental surgeon.

This is undoubtedly an exciting time in dentistry and the biomedical community at large. In 20 to 25 years, dentistry, as we know it today, will be remarkably different, as different as it is now, compared to what it was 25 years ago. Many dental colleges and postgraduate programs are currently evaluating curriculum content in the light of the oral health care needs of the public and in the light of many advances in genetics, cell and molecular biology, and materials sciences. At the pre-doctoral level, tissue engineering provides an ideal opportunity to incorporate a multi-disciplinary learning experience into the curriculum, which integrates concepts in cell biology, molecular biology, bio-engineering and biomaterials with clinical techniques in oral surgery, periodontics, restorative dentistry, and oral medicine. Students can see first-hand the interplay between the science underlying tissue engineering and its clinical application to oral disease. Such an experience would also allow students to see collaboration among biomedical scientists, dentists, and physicians, a phenomenon that is extremely rare in most dental college programs. At the postgraduate level, there is a need to provide the community with a cadre of M. D. S. trained practitioners, researchers, and educators, with expertise in tissue engineering. For the practitioner, continuing education programs can increase the awareness of tissue engineering as a therapeutic option for various oral health problems. These programs can also help establish linkages between dentists in the community and tissue engineering specialists at academic health centers. Once the general public is aware of newer and better treatments, they will not accept anything less. A well-informed clinician, capable of incorporating this technology into his or her practice, will continue to thrive in the future.

